# Structural Analysis of How Podocytes Detach from the Glomerular Basement Membrane Under Hypertrophic Stress

**DOI:** 10.3389/fendo.2014.00207

**Published:** 2014-12-12

**Authors:** Wilhelm Kriz, Brunhilde Hähnel, Hiltraud Hosser, Sigrid Rösener, Rüdiger Waldherr

**Affiliations:** ^1^Institute of Transfusion Medicine and Immunology, Medical Faculty Mannheim, University of Heidelberg, Mannheim, Germany; ^2^Institute of Neuroanatomy, Medical Faculty Mannheim, University of Heidelberg, Mannheim, Germany; ^3^Pathologie Heidelberg, Heidelberg, Germany; ^4^Global Non-Clinical Safety, Merck KGaA, Merck Serono, Darmstadt, Germany

**Keywords:** podocyte hypertrophy, foot process effacement, sealing of filtration slits, pseudocysts, podocyte detachment, crescents

## Abstract

Podocytes are lost by detachment from the GBM as viable cells; details are largely unknown. We studied this process in the rat after growth stimulation with FGF-2. Endothelial and mesangial cells responded by hyperplasia, podocytes underwent hypertrophy, but, in the long run, developed various changes that could either be interpreted showing progressing stages in detachment from the GBM or stages leading to a tighter attachment by foot process effacement (FPE). This occurred in microdomains within the same podocyte; thus, features of detachment and of reinforced attachment may simultaneously be found in the same podocyte. (1) Initially, hypertrophied podocytes underwent cell body attenuation and formed large pseudocysts, i.e., expansions of the subpodocyte space. (2) Podocytes entered the process of FPE starting with the retraction of foot processes (FPs) and the replacement of the slit diaphragm by occluding junctions, thereby sealing the filtration slits. Successful completion of this process led to broad attachments of podocyte cell bodies to the GBM. (3) Failure of sealing the slits led to gaps of varying width between retracting FPs facilitating the outflow of the filtrate from the GBM. (4) Since those gaps are frequently overarched by broadened primary processes, the drainage of the filtrate into the Bowman’s space may be hindered leading to the formation of small pseudocysts associated with bare areas of GBM. (5) The merging of pseudocysts created a system of communicating chambers through which the filtrate has to pass to reach Bowman’s space. Multiple flow resistances in series likely generated an expansile force on podocytes contributing to detachment. (6) Such a situation appears to proceed to complete disconnection generally of a group of podocytes owing to the junctional connections between them. (7) Since such groups of detaching podocytes generally make contact to parietal cells, they start the formation of tuft adhesions to Bowman’s capsule.

## Introduction

Podocyte loss underlies the progression of glomerular diseases to end-stage renal failure, both in human beings (1) and in experimental animal models (2). How podocytes are lost is open to debate. Apoptosis is widely considered as a major mechanism, but this has been demonstrated largely in cell culture, and convincing evidence showing apoptosis of podocytes *in situ* by transmission electron microscopy (TEM) has never been presented. Also, necrosis of podocytes has rarely been shown and if it occurs, it is mostly in cells already detached from the GBM. On the other hand, viable podocytes in the urine have been found in several glomerular diseases (3, 4) and podocytes appearing to be viable detaching from the GBM in huge numbers were shown by TEM in various experimental models (5). This leads to the conclusion that detachment as viable cells is the major mechanism of podocyte loss and that the major causes of podocyte loss are likely to be mechanical forces challenging the attachment of podocytes to the GBM (6).

Recently, the hypothesis has been raised that the shear stress derived from filtrate flow through the filtration slits acting on the foot processes (FPs) may have a major impact on podocyte detachment (6, 7). Moreover, it has been suggested that glomerular hypertension, hyperfiltration, and excessive hypertrophy, which have previously been shown to underlie progression of glomerular diseases (8, 9), may account for increases in shear stress compromising the attachment of podocytes to the GBM.

Furthermore, it has been postulated that major changes of podocytes in glomerular diseases, such as foot process effacement (FPE) and cytoplasm shedding, which have been generally interpreted as pathological derangements of podocytes, actually show the “podocyte’s fight” against detachment, and thus are changes undertaken to secure survival, i.e., continued attachment to the GBM (5).

The present study analyzes the individual steps of podocyte detachment in a model of excessive glomerular hypertrophy that had been previously studied by our group (10). Daily administration of FGF-2 led to a long-term growth stimulation of glomeruli followed by the development of widespread focal and segmental glomerulosclerosis (FSGS). At the time of this study, in 1995, the idea that podocytes are lost as viable cells in the urine was not yet born. The focus of the study lay in the question of how, subsequent to podocyte loss, tuft adhesions develop and lead to the degeneration of the entire nephron. In the present study, we used the same material to structurally analyze earlier stages to describe how podocytes undergo detachment. We show that the process of detachment from the GBM is extremely gradual and frequently complicated by the fact that the sequence of steps to detachment may be mixed up with changes locally reinforcing the attachment to the GBM.

## Materials and Methods

The material used in the present study was derived from previous experiments (10). Therefore, only a brief description of the methods is given here. The experiments complied with the “German law on the protection of animals.”

A total of 36 Sprague-Dawley rats (7 weeks old; half males, half females) were studied. Half received daily subcutaneous injections of 320 μg FGF-2/kg body weight and the other half served as controls, receiving the vehicle only. Two-thirds of the rats were studied after total body perfusion at 8 weeks; the remaining one-third was studied after 13 weeks. After anesthesia with Nembutal^®^ (1.2 ml/kg body weight), the kidneys were retrogradely perfused via the abdominal aorta without prior flushing for 3 min at a pressure of 220 mm Hg. In 24 rats intended for ultrastructural studies, the fixative contained 1.5% glutaraldehyde and 1.5% formaldehyde in phosphate buffered saline (260 mOsm, pH 7.4) supplemented with 0.5 g/l picric acid. Blocks of cortical tissue were processed by standard procedures (i) for paraffin sections (4 μm; stained with hematoxylin eosin) and (ii) after postfixation with OsO_4_ or tannic acid (11), for Epon sections (1 μm, stained with methylene blue) to be studied by light micrograph and ultrathin sections stained with uranyl acetate and lead citrate to be studied by TEM. The remaining 12 rats were perfused with paraformaldehyde [see Ref. (10)].

### Structural studies

**Figure 1:**
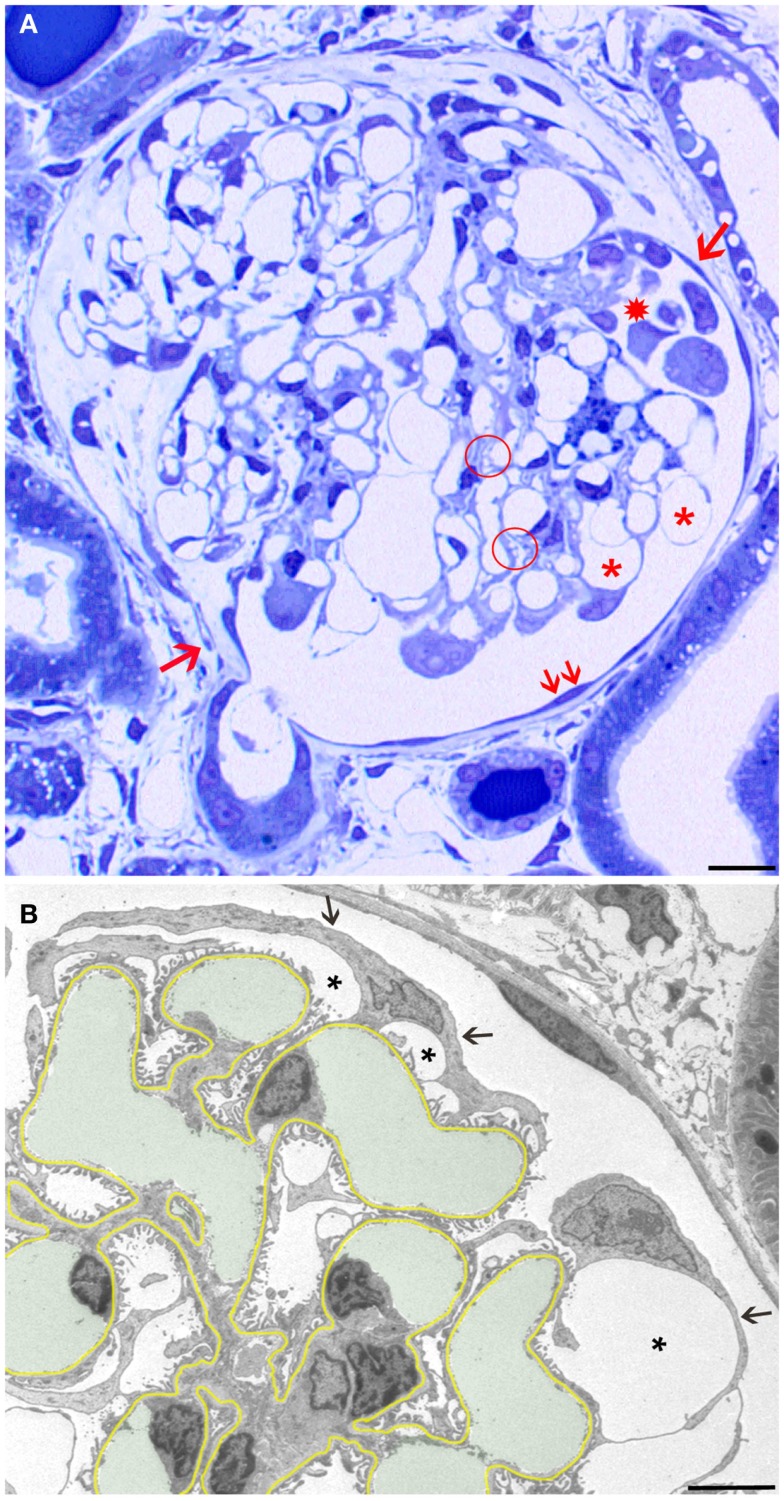
**Overview and early changes**. **(A)** Light micrograph (LM) of a glomerular profile showing the crucial injuries presented later in more detail in transmission electron micrographs (TEMs) and quantified in Table 1. Hypertrophied podocytes forming large pseudocysts (asterisks), areas of small pseudocysts generally associated with local detachments (enclosed by a hatched circle), detachment of podocytes forming a cluster in Bowman’s space (star), and a large tuft adhesion comprising almost two-thirds of the tuft (clockwise from arrow 1 to arrow 2) are seen. Note that outside the tuft adhesion, the parietal epithelium looks fully normal (two small arrows). **(B)** The GBM is highlighted in yellow, capillary lumens in green. The cell bodies of podocytes are attenuated (arrows) and pseudocysts (asterisks) have developed underneath them. Note that the foot process pattern is widely preserved including the floors of pseudocysts and, second, the parietal epithelium looks fully normal. Male rats, growth stimulation with FGF-2 for 13 weeks. **(A)** LM from 1 μm Epon section. Bar: 15 μm. **(B)** TEM. Bar: 5 μm.

In addition to the structural assessments in the previous study (10), a quantification of glomerular injuries as defined in the present study (pseudocysts, podocyte detachment associated with small pseudocysts, podocyte clusters, and tuft adhesions; Figure 1A) was done. In three independent 1 μm Epon sections of each experimental animal (6 males and 6 females; values from 8 and 13 weeks were pooled) and of six controls, the glomerular profiles and the number of the above listed lesions found in these glomeruli were counted, figured up for each animal, expressed as percentage of the total number of profiles of each animal, and compared. In order to receive an overall weighted damage index, we graded large pseudocysts with 1, accumulations of small pseudocysts with 2, clusters of podocytes with 3, and adhesions with 4. Results are reported as mean ± SD; differences between the various groups were tested by one-way ANOVA using Sigma Plot.

## Results

Essential stereological results of the previous paper (10) are summarized as follows. Prolonged daily treatment of rats with FGF-2 for 8 weeks led to increasing albuminuria (0.30 ± 0.15 versus 141.5 ± 132.4 mg/18 h) in males and (0.11 ± 0.02 versus 79.8 ± 150.3 mg/18 h) in females; the respective numbers after 13 weeks read 0.21 ± 0.09 versus 303.4 ± 78.4 and 0.13 ± 0.03 versus 85.1 ± 31.5. Serum creatinine increased significantly, indicating the development of chronic renal failure. No increase in body weight or in kidney weight compared with controls was found. However, a prominent significant increase in glomerular tuft volume (1651 ± 209 versus 3035 ± 596 μm^3 ^× 10^3^ in males, 1138 ± 85 versus 1998 ± 392 μm^3 ^× 10^3^ in females), and a numerical increase in mesangial and endothelial cells but no increase in podocyte number were found, resulting in a dramatic decrease in podocyte density (N podo/μm^2 ^× 10^3^), more pronounced in males (0.86 ± 0.007 versus 0.48 ± 0.07) than females (1.05 ± 0.09 versus 0.67 ± 0.14).

The discrepant growth of glomeruli with podocytes growing by hypertrophy only, resulted in structural changes that terminated in the detachment of podocytes from the GBM. The present study focused on the structural details of how podocytes reinforce attachment to the GBM, undergo detachment from the GBM and on the fate of detached podocytes.

The first derangements of podocytes consisted of cell body attenuation and pseudocyst formation (Figure 1B), which, as shown previously in another model (12), were likely causally linked. Growing and expanding capillaries forced podocytes to cover increased surfaces, leading to stretching and broadening of podocytes’ cell bodies. This, in turn, resulted in narrowing of the outflow clefts from the subpodocyte spaces, causing the focal bulging of the attenuated cytoplasmic sheets, usually called pseudocysts. Of note, at this stage, podocytes generally showed a normal FP pattern that also covered the floor of pseudocysts (Figure 1B).

The structural changes that finally led to the detachment of podocytes clearly started after this stage at the FPs (not necessarily as a consequence). The description is complicated by the fact that clear signs of detachment are mixed up with structural responses of podocytes taken to counteract detachment, which are generally subsumed under the term “FPE” (5).

**Figure 2:**
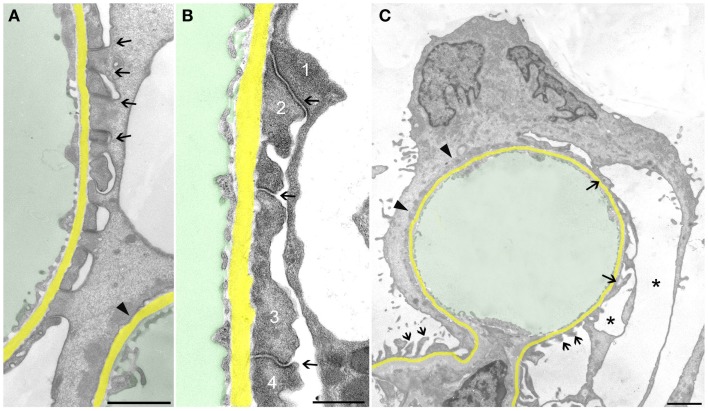
**Sealing of the slits may proceed to complete foot process effacement (FPE)**. The GBM is highlighted in yellow, capillary lumens in green. **(A)** Early stage of FPE with broadening of FPs and sealing of the slits by occluding junctions (some are labeled by arrows). In the right upper corner part of a capillary, profile with completed FPE (arrowhead) is seen. **(B)** “Foot processes” have changed into broad plaques (1,2,3,4) that closely stick to the GBM. The sealing of the filtration slits by occluding junctions between them is maintained (arrows). **(C)** Capillary profile covered by a podocyte that is closely attached to the GBM by “effaced” basal cell portions stuffed with a prominent cytoplasmic mat (arrowheads). At the opposite side, broadened FPs (arrows) are undergoing retraction, whereas at the transition to the mesangial region, FPs appear intact (two small arrows). Pseudocyst formation is also seen (asterisks). **(A)** and **(B)** male, **(C)** female rats, growth stimulation with FGF-2 for 13 weeks. TEMs. Bars: **(A)** 1 μm, **(B)** 0.5 μm, **(C)** 2 μm.

Let us first consider the changes that may be suggested to be protective, i.e., to counteract detachment. FPs broadened making the filtration slits narrower; finally, the slit diaphragms were replaced by occluding junctions, thus sealing the filtration slits (Figure 2A). This was associated with a retraction of FPs into short, irregularly shaped cell projections (Figure 2B). Further on, the development led to the completion of FPE, changing podocyte processes into broad, flattened, disk-like projections that finally fused with the cell bodies resulting in podocytes adhering to the GBM along their total basal aspect. Thus, podocytes had become largely separated from each other, meaning that individual podocytes covered a clearly delimited capillary area, frequently the total circumference of one capillary profile (Figure 2C). Concomitantly, in the basal parts of these podocytes, a microfilament-based cytoskeletal mat developed (13) that has been interpreted as a way to achieve stronger adherence of podocytes to the GBM (5). This appearance may be regarded to underlie the successful prevention of podocyte detachment.

**Figure 3:**
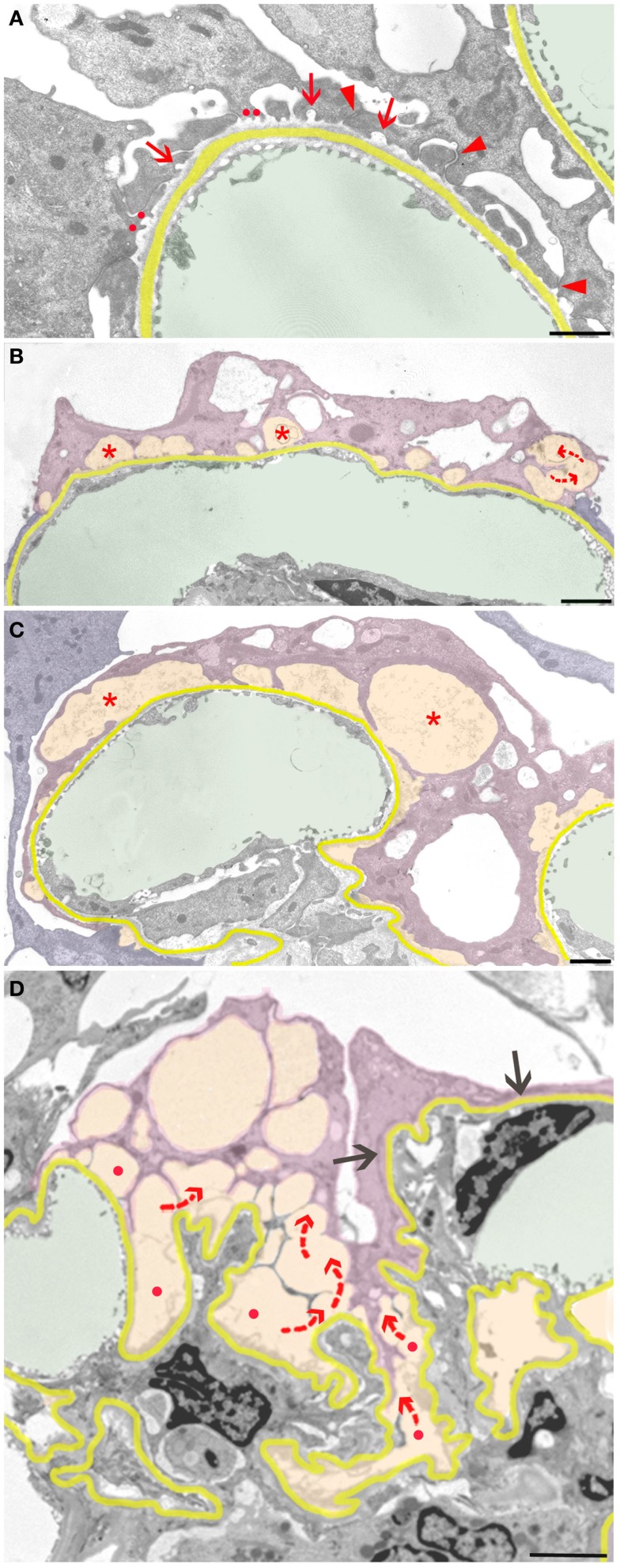
**Sequence of podocyte disconnection from the GBM**. The GBM is highlighted in yellow, capillary lumens in green, and spaces considered as pseodocysts connected to bare GBM in yellow-orange. Individual podocytes are highlighted in different shades of violet. **(A)** Early stage of podocyte detachment from the GBM. A sealing of filtration slits by occluding junctions replacing slit membranes was only partially successful (arrowheads). At many other sites, retraction of FPs has left behind circumscribed areas of bare GBM (arrows) that start to merge (red dots). **(B)** Advanced stage of podocyte detachment from the GBM. The dome-shaped spaces (asterisks) above bare areas of GBM have enlarged bulging toward the urinary space. Merging of these spaces leads to a communicating system of pseudocysts (arrows). **(C)** The pseudocysts (asterisks) increase in size by merging and bulging; they are related to large stretches of bare GBM and overarched by broadened and attenuated primary processes. **(D)** The final detachment of a podocyte is frequently characterized by the merging of innumerous interconnected (red arrows) pseudocysts that start over bare GBM (red dots) and drain into Bowman’s space (not seen in this section). Other portions of this podocyte may closely stick to the GBM with completed FPE (black arrows). Male rats, growth stimulation with FGF-2 for 13 weeks. TEMs. Bars: **(A)** 1 μm, **(B)** 2 μm, **(C)** 1 μm, **(D)** 2.5 μm.

Neighboring such developments, even at other aspects of the same podocyte, these protective responses may fail, starting the focal detachment of podocytes at seemingly random sites. The complete detachment of an entire podocyte results from the final coalescence of numerous discrete disconnected areas (Figure 3). The smallest and probably first signs of detachment consist of circumscribed bare areas of GBM encountered in between two FPs of the same podocyte, thus the former interposed FP was missing (Figure 3A). This phenomenon frequently occurs in series and may be derived from the retraction of a series of FPs arising from a common larger process. In such cases, the co-ordinated retraction of adjacent FPs after formation of occluding junctions between them (as described above) seems to have failed.

Those areas of circumscribed detachments are frequently overarched by dome-shaped, flattened podocyte processes that are still attached to the GBM (Figure 3B). Thus, a new kind of small pseudocyst has developed that – in contrast to the above-described large pseudocysts – is underlain by sections of bare GBM. Progressive local detachment leads to the coalescence of these spaces (Figure 3C) and, finally, also to coalescence with large pseudocysts, creating a communicating system of extracellular spaces, through which the filtrate has to pass to reach Bowman’s space (Figure 3D). Podocytes associated with such assemblies of pseudocysts finally undergo detachment.

**Figure 4:**
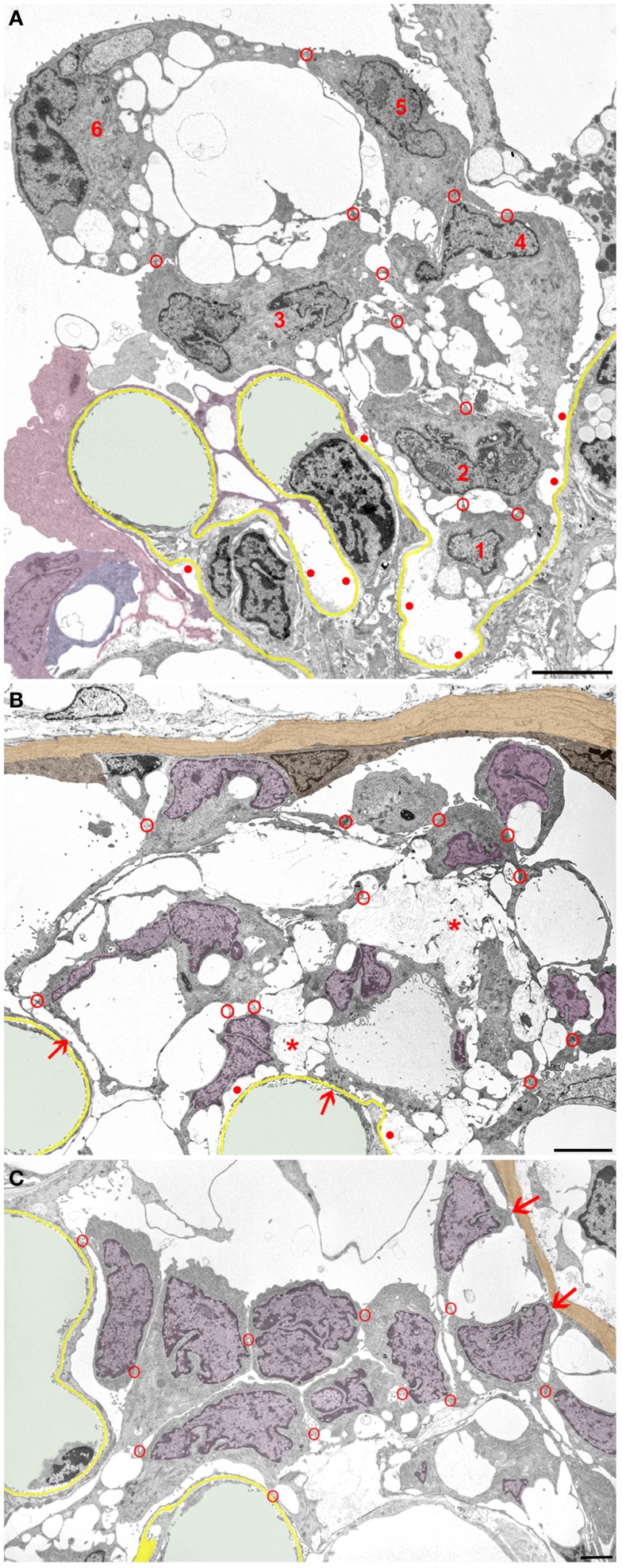
**Clusters of detaching podocytes in Bowman’s space**. Capillary lumens are highlighted in green, the GBM in yellow. In B and C cell nuclei of podocytes are shown in violet, the PBM in light brown. **(A)** A group of 6 podocytes in the process of detachment. Only podocytes 1 to 4 still have contact with the GBM, whereas 5 and 6 are attached to the podocytes beneath by junctions (red circles). Within this cluster of podocytes, a communicating system of pseudocysts is seen that starts at bare areas of GBM (red dots). It may readily be suggested that the filtrate entering through bare GBM has to pass these spaces to reach Bowman’s space; openings into Bowman’s space are not hit in this section. Note that additional detaching podocytes (colored in various shades of violet) are gathered in another cluster. **(B)** Cluster of some 10 detaching or detached podocytes forming a cell bridge between the tuft and Bowman’s capsule. The unlabeled cell within the parietal epithelium cannot be assigned by any plausible criterion to either parietal cells or to podocytes. The innermost cells are still partially attached to the GBM (arrows). The cells are interconnected to each other and still contain abundant pseudocysts that form a continuous chamber system starting from bare GBM (red dots) extending to the outermost layer. Note that the walls of many pseudocysts are undergoing shedding (asterisks). **(C)** Podocytes after almost complete detachment from the GBM are assembled as a cluster in Bowman’s space interconnected to each other by cell contacts (red circles). The inner row of podocytes shows assemblies of small pseudocysts, the outer row appears as rounded cells. The outermost cells have contacts to Bowman’s capsule (arrows). Male rats, growth stimulation with FGF-2 for 13 weeks. TEMs. Bars: **(A)** 5 μm, **(B)** 4 μm, **(C)** 2 μm.

In general, podocytes detach in groups (Figure 4) forming clusters of cells within Bowman’s space, cells interconnected by occluding junctions. These interconnections are likely rooted in the early response to the danger of detachment, i.e., sealing the slits by replacing the slit diaphragms by occluding junctions. As mentioned above, sites of detachment from the GBM and sites of reinforced attachment including the attachment to adjacent podocytes may be found in the same podocyte early in the disease process. These interconnections were, at least partially, preserved when podocytes finally detached from the GBM as entire cells.

**Figure 5:**
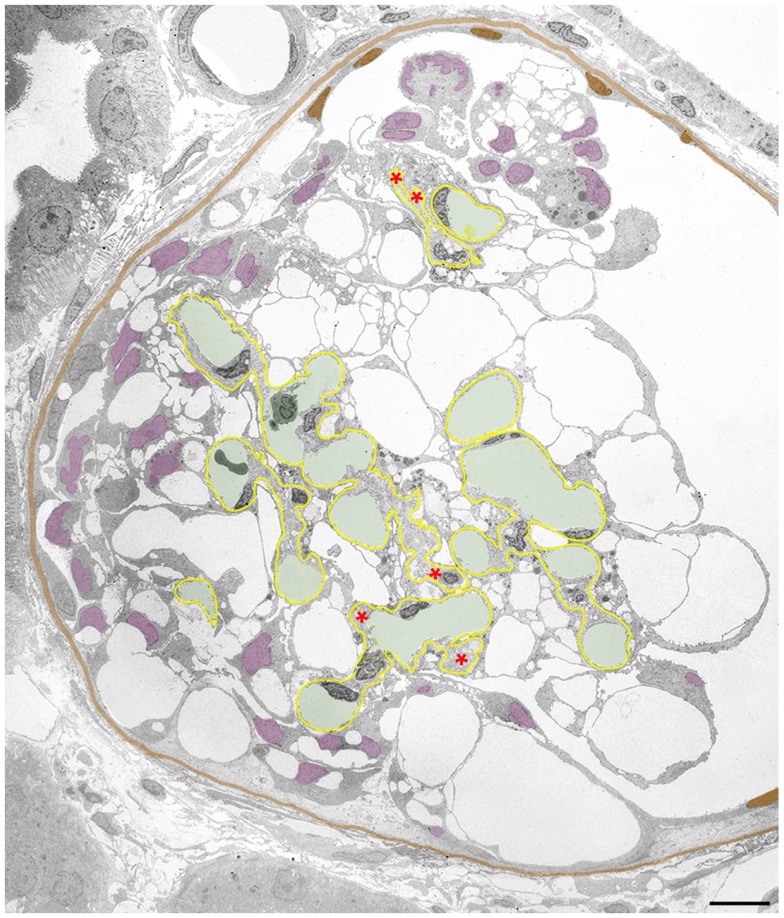
**Clustering of podocytes in Bowman’s space is associated with shrinkage of the mesangio-capillary area**. The GBM is highlighted in yellow, capillary lumens in green, the PBM and cell nuclei of parietal cells in brown, podocyte cell nuclei in violet. Glomerular profile with a tuft that is broadly connected to Bowman’s capsule by a large cellular crescent that surrounds the tuft from three sides. The mesangio-capillary area (delimited by the GBM) is small compared to the area occupied by podocytes. Note that podocyte cell bodies (visualized by their cell nuclei) have almost completely disappeared from central tuft areas being all contained within the crescent. Most of the podocytes have retained pseudocysts. The cluster of podocytes on top of the tuft does not show any contacts to the tuft nor to the PBM, whereas the major part of the crescent displays extensive contacts to the GBM as well as to Bowman’s capsule. Nuclei of cells, which by no plausible criterion can be assigned as podocytes or parietal cells are uncolored. Note that the GBM is heavily wrinkled at several sites and the mesangial area appears to be collapsed with disappearance of capillaries at several loci (asterisks). Male rat, growth stimulation with FGF-2 for 13 weeks. TEM. Bar: 10 μm.

Initially, those clusters of podocytes are connected to the GBM by the innermost cells (Figure 4A). In later stages, they made contact via the outermost cells with the parietal epithelium (Figures 4B,C and 5), thereby forming multicellular “bridges” between the GBM and the parietal epithelium. During this process, podocytes change in shape. Apparently by shedding of the pseudocysts, i.e., of their thin walls, the detached podocytes turned into groups of rounded cells without any matrix in between (Figures 4 and 5). Mitotic figures were never encountered in these cells.

In even later stages, these bridging clusters of detached podocytes initiate the formation of tuft adhesions to Bowman’s capsule, i.e., firm cell and matrix connections between the GBM and the parietal basement membrane, as described previously (10). Concomitantly, the tuft itself underwent considerable shrinkage, including wrinkling of the GBM, loss of capillaries, and mesangial cells (Figure 5; to be described in detail in another context).

The detachment of podocytes that have come to protrude into the urinary orifice has already been described in various models and interpreted as the consequence of high shear forces within the urinary orifice acting on the cell bodies (5, 14). Here, we found that the final changes accounting for detachment are likely also rooted in the destabilization of FP attachment to the GBM, as described above, and that these podocytes frequently detach in groups and travel in groups along the tubule (not shown).

### Quantification of glomerular injuries

Crucial steps of the above-described sequence in damage progression were quantified in 1 μm sections. Figure 1A shows an entire glomerular profile indicating the injuries that were counted and compared. As seen in Table 1 and in agreement with the clinical data of the former study (10), the damage was significantly more severe in males than in females.

**Table 1:** **Quantification of glomerular injuries**.

	Experimental animals		Controls
	
	8 + 13 weeks, pooled		
	Female, *n* = 6	Male, *n* = 6	*n* = 6
No. of glomerular profiles	536	385	509
Large pseudocysts%	77 ± 11	87 ± 7	0
Small pseudocysts%	37 ± 8*	62 ± 16	0
Clusters of podocytes%	19 ± 9**	42 ± 7	0
Tuft adhesions%	4 ± 3*	18 ± 21	0
Weighted damage score	56 ± 30**	103 ± 48	0

*A glomerular profile was considered positive for the respective injury if a minimum of (i) three large pseudocysts, (ii) two areas of small pseudocysts (indicative of local detachments), (iii) four podocytes forming a cluster of connected cells in Bowman’s space, and (iv) one adhesion was encountered. The weighted damage score includes all changes in a graded way (see Materials and Methods)*.

*p < 0.05, **p < 0.001.

## Discussion

### General

It is commonly agreed that the loss of podocytes underlies the development of progressive renal failure. Research of the past 20 years has shown that podocytes are lost by a unique mechanism, i.e., by detachment from the GBM as viable cells and excretion in the urine. This fact, in conjunction with their inability to replicate, puts podocytes in a precarious situation, unlike any other cell (5). Thus, the precise knowledge of why and how podocytes undergo detachment from the GBM is of great importance when searching for strategies to delay the progression to end-stage renal failure.

The present study has addressed this question in a model of excessive glomerular growth initiated by long-term stimulation with FGF-2 (10). Endothelial and mesangial cells proliferated in this condition; podocytes participating solely by hypertrophy could not cope with the overall glomerular growth, underwent structural derangements and, finally, detached from the GBM. This growth-stimulating rat model is similar to a recently published transgenic rat model (15) in which the ability of podocytes for hypertrophy was restricted resulting, after growth stimulation by uninephrectomy, in a mismatch of endothelial and mesangial cell proliferation on the one hand and podocyte hypertrophy on the other, followed by podocyte loss through detachment. Both models mirror what happens in degenerating progressive glomerular diseases including all forms of secondary FSGS.

### The detachment of podocytes from the GBM

The difficulty in the interpretation of the changes encountered in conjunction with podocyte detachment consists of separating the sequence of changes progressing to detachment from the sequence terminating in complete FPE, thus reinforcing attachment to the GBM. Changes belonging to either of these developments may be found at different sites of the same podocyte. This emphasizes the most important insight of this study: podocyte damage is local, even in the sense that the same podocyte undergoes detachment in one part of the cell, whereas at another part, it successfully reinforced its attachment to the GBM. This fact supports the view that, at least in this model, overall derangements of podocyte cell function are not the crucial causes underlying detachment but local imbalances between the forces accounting for attachment and those for detachment appear to play the major role.

This triad of hypertrophy, hypertension, and hyperfiltration has been convincingly shown to account for the progression of glomerular diseases under a great variety of circumstances (8, 9). These three factors are causally interconnected with each other but it remains an open question whether one of them plays a dominant role. Likely all three are involved in a mutually reinforcing process (16, 17). An elegant study showing in two mouse models of glomerulosclerosis that the development of glomerular injuries was significantly curtailed after unilateral ligation of the ureter emphasizes the effects of changes in filtration dynamics (18), whereas prevention of glomerular growth in a model with established hyperfiltration and glomerular hypertension also resulted in a marked decrease in sclerosis development (19), showing a major impact of growth. Also, in human beings, glomerular hypertrophy was found to be the decisive cofactor in minimal change disease accounting for progression to FSGS (20). The present study suggests that excessive hypertrophy of podocytes may be a major factor in progression to what extent hyperfiltration and glomerular hypertension develop in this model is unknown.

Therefore, damage development in the FGF-2 model may be taken to reflect the combined effects of all three factors.

Let us look in detail to a hypothetical local scenario that may start the process of detachment. A certain podocyte has reached its ultimate limit of hypertrophy that allowed it to cover a correspondingly increased area of GBM with normal FPs. In case the rheological situation with high filtrate flows overtaxes the available slit area, the only protective option remaining consists of sealing the slits by occluding junctions. This represents a most crucial event. The formation of cell–cell junctions between FPs in glomerular disease models has been described previously by several groups, characterizing them as an “occluding” type (21–27). More recent work has shown that they are a certain type of tight junctions (28, 29). Moreover, it was shown that tight junctional proteins increase several-fold in puromycin-induced nephrosis (29).

If sealing the slits is successful, compensation by adjacent podocytes is required, with these being exposed to higher local filtrate flows. If these podocytes have some reserve capacity to hypertrophy, they may adequately manage the situation.

In the case that the sealing strategy fails, a fatal situation will develop. Damage to the slit membrane will increase the shear stress on the involved FPs and may initiate its local detachment, thereby increasing the bare area of GBM. This will lead to local increases in flow (local hyperfiltration) and worsen the unstable situation. Thus, as soon as any detachment has started locally, the final struggle against the detachment of the entire cell has begun. If there is any chance to reverse this situation, it probably consists of spreading of neighboring, uninvolved podocytes on the GBM covering the denuded area. If this maneuver also fails, detachment of the first podocyte will proceed and may include adjacent podocytes.

The process of detachment seems to follow a rather stereotyped structural sequence. Circumscribed bare areas of GBM in between two adherent FPs of the adjacent podocyte (as the result of initial local detachments) frequently form the floors of dome-like cavities overarched by attenuated cytoplasm of broadened primary processes. These spaces may be considered as a second type of pseudocyst associated, in contrast to the large pseudocysts, with bare GBM. They merge with each other, later with the large pseudocysts, finally forming a communicating system of extracellular spaces that starts above scattered areas of bare GBM and opens into Bowman’s space through the narrow openings of the subpodocyte spaces. In many previous studies, this system of empty spaces has been termed “podocyte vacuolation,” considered to consist of intracellular vacuoles (“blebs”) (23, 30–34), indicating a prenecrotic stage. Here, we show that for the most part, these are extracellular spaces bordered by intact cell membranes of viable podocytes.

It may readily be suggested that sites of bare GBM allow increased filtrate flows and are the sites of protein leakage, as frequently suggested and shown previously (22, 35–37). The filtrate has to find a way out of these spaces along routes offering some amount of resistance to the outflow. The resulting expansile forces on the walls of these spaces induce progressive bulging and detachment, leaving behind increasing areas of bare GBM. Consequently, the flow of the filtrate through the system of connected pseudocysts likely exerts expansile forces on the concerned podocyte, tending to lift it off the GBM (Figures 4 and 5).

Even if started locally at a single podocyte, the process of detachment generally comprises a group of podocytes. This may be another explanation for the observation by Ichikawa et al. (38) that damage to a podocyte always damages neighboring podocytes. In our view, the detachment of a podocyte, once started, will often take others along and may be difficult to arrest.

The interpretation of the sealing of filtration slits by “FPE” as a protective measure of podocytes against detachment conflicts with the widely held view that FPE represents the common initial derangement of podocytes leading to proteinuria potentially progressing to more severe glomerular damage (39). As discussed previously (5), FPE is necessarily accompanied by transient protein leakage as a consequence of the motility associated with the extensive local rearrangements. The transient character of this kind of proteinuria has recently been found in studies after Rac-1 stimulation (40–42) that is known to elicit FPE (39, 43). Progression of the damage to FSGS has not been found in these studies supporting our view of a protective impact.

### The fate of detached podocytes

In static structural studies, it is impossible to follow individual podocytes as they are translocated from one site to another. However, we can observe that along the course of the disease, podocytes are regularly encountered at other sites than where they are found under normal conditions. From such comparisons, we may deduce the routes how they came from site A to site B. We do not think that such movements of podocytes are effected by any active motility of the podocytes themselves, We think that detachment from the GBM exposes them to the rheological forces of filtrate flow pushing them toward Bowman’s capsule and the urinary orifice.

The fraction of podocytes that detach directly in front of the urinary orifice generally will be swept into the tubular lumen (5, 10, 14). Surprisingly, during their passage along the tubule, they did not undergo anoikosis but, as known from several studies (e.g., 3 and 4), may have reached the renal pelvis as living cells.

Podocytes detaching from most other sites of the tuft may form massive cell accumulations within Bowman’s space interconnected by occluding junctions with the innermost cells keeping contact to the GBM. During this gradual process of detachment, podocytes change in shape; they shed all the peripheral material forming the thin walls of pseudocysts, finally appearing as rounded cells. Frequently, such cell clusters establish, with their outermost cells, contacts to parietal epithelial cells, thus forming purely cellular crescents connecting the tuft and Bowman’s capsule.

These observations are in full agreement with recent *in vivo* studies by multiphoton microscopy in two other models with podocyte detachment, i.e., unilateral ureter ligation (UUO) and Adriamycin nephropathy (44). In these studies, the formation of multicellular clusters of podocytes in Bowman’s space was observed that made contact to Bowman’s capsule; also, podocytes in single file moving down the tubule were seen. The authors also observed that the podocyte clusters made contacts to parietal cells by cell projections and nano-tubes. We found by TEM abundant occluding junctions between podocytes as well as between podocytes and parietal epithelial cells suggesting that they are responsible for the mechanical cohesion. As also verified in serial sections, the outermost cells of the podocyte clusters are fully separated from the GBM but fixed to inner cells of the cluster by occluding junctions. It appears that as soon as they come into contact with parietal cells, they establish junctional contacts to parietal cells, as well.

The further evolution of such clusters of detached podocytes attaching to Bowman’s capsule, finally turning into tuft adhesions and crescents has been shown and discussed previously (10, 45, 46).

#### Relationships to pathologies seen in human renal diseases

It is quite obvious that the clustering of podocytes within Bowman’s space and the shrinkage and collapse of the mesangio-capillary area have equivalents in human renal diseases, known as “cellular crescent,” “cellular lesion,” and “collapsing glomerulopathy” (CGP). The origin of the abnormal cells in these pathologies is under debate (47–50). In the present degenerative animal model, the cells clustering on the outer aspect of the tuft within Bowman’s space are clearly derived from podocytes and do not proliferate. CGP combines the collapse of the mesangio-capillary compartment with hypertrophy and seemingly hyperplasia of the cells that take the place of podocytes on the outside of the GBM, assembled in clusters looking like crescents (51–54). The shrinkage of the mesangio-capillary area with wrinkling of the GBM and collapse of capillaries in CGP look identical to what is seen in the present animal model. Also, the clustering of cells in Bowman’s space in CGP may look very similar to the clustering of detached podocytes in our animal model.

The great number of such cells seen in a cross-sectional profile of CGP seems incompatible with detachment as the only mechanism. However, as far as we know, the total number of podocytes on the surface of a collapsed tuft in CGP has never been estimated by stereological methods. Though a fully reliable calculation is presently not possible, we tried the following approach applying the recently published method by Venkatareddy et al. (55) to estimate podocyte number from a single glomerular profile in a single section. Since we do not have own biopsy material from a case of CGP, we took the glomerulus shown in Figure 3 from the paper by Jhaveri et al. (56) and made the following calculation. In this glomerular profile, we counted 90 podocyte cell nuclei. From the listed magnification, the tuft diameter is about 100 μm (healthy tufts are usually in the range 180–220 μm, glomerular tufts are shrunken in CGP). The cross-sectional area is, therefore, 7854 μm^2^ and the area density of nuclei is 90/7854 = 0.01146. Using the estimated caliper diameter for human podocyte nuclei of 8.2 μm (given in 55), we obtain the volumetric podocyte density of 0.01146/8.2 = 0.001397 podocytes/μm^3^. The 100 μm tuft diameter yields an estimated tuft volume of 523,585 μm^3^ (much smaller than a typical tuft volume of 3,000,000 μm^3^). Multiplying the density by the tuft volume gives an estimate of 731 podocytes in the tuft. To the degree that any podocytes are binucleate, this will be an overestimate of cell number.

Thus, it seems possible that the seemingly great number of podocytes on a shrunken tuft in CGP is all derived from detachment, gathering on the shrunken surface without any cell proliferation. This is supported by the fact that mitotic figures are generally not found in these cell assemblies in CGP. Major support in favor of detachment comes from the work of Bariety et al. (57) and also from findings in a transgenic mouse model of HIV-associated nephropathy (58).

## Conclusion

Loss of podocytes represents the central event underlying the glomerular disease progression (4, 46). Here, in a model of continuous growth stimulation by FGF-2, we show that loss of podocytes occurs by detachment from the GBM as viable cells. This is a gradual process that starts focally at randomly distributed sites. The local origin strongly supports the view that the forces starting detachment consists of local rheological disturbances. The detachment of a podocyte, once started, may be difficult to arrest. However, podocytes have developed protective mechanisms against detachment, most important the sealing of the filtration slits followed by FPE.

## Conflict of Interest Statement

The authors declare that the research was conducted in the absence of any commercial or financial relationships that could be construed as a potential conflict of interest.

## Abbreviations

CGP, collapsing glomerulopathy; FP, foot process; FPE, foot process effacement; FSGS, focal segmental glomerulosclerosis; GBM, glomerular basement membrane; PBM, parietal basement membrane.
